# The initiation of the second-step intradisciplinary tumor board discussion and its impact on treatment decision. Retrospective data analysis of 12 years’ experience in a tertiary oncology center

**DOI:** 10.3389/fonc.2025.1553874

**Published:** 2025-08-12

**Authors:** László Csaba Mangel, Erika Kövér, Balázs Pécsi, Imre Boncz

**Affiliations:** ^1^ Institute of Oncotherapy, Medical Faculty and Clinical Center, University of Pécs, Pécs, Hungary; ^2^ Head Office of Clinical Center, University of Pécs, Pécs, Hungary

**Keywords:** cancer care, tumor board, multidisciplinary, intradisciplinary, treatment decision

## Abstract

**Background:**

Multidisciplinary team (MDT) meetings are generally accepted forums for the quality of cancer care, however, there is an ongoing discussion about the substantial role of MDTs in reaching optimal treatment decisions. In our tertiary oncology center, a second-step intradisciplinary seu oncotherapy tumor board (OTT) discussion system was introduced to increase the adherence of MDT’s decision making with the knowledge of patients’ preference and tolerance, and to partially relieve MDT’s overwork in the purely adjuvant and the palliative treatment settings. Over the real-world tumor board data elaboration, the primary aim of this observational study was to present the impact of OTT meetings on treatment decisions.

**Methods:**

The data of 33,056 cases of 27,227 patients were retrospectively analyzed with using a regular expression-based word search algorithm. Subsequent modifications of OTT decisions were defined as “minor”, when only some additional suggestions were introduced, “moderate” when the treatment items were significantly modified, and “major” when the direction of the treatment was fully transformed.

**Results:**

During the 12-year observation period (2007-2019) the number of patients and case discussions, average age of the patients, percentage of sophisticated treatment methods, and the number of treatment lines/decisions made for the same patient had been continuously increased. The average percentage of minor, moderate and major modifications were 2.28, 6.4 and 8.92%, respectively, implying a remarkably high modification rate of the primary recommendations.

**Conclusion:**

Considering the growing complexity and multiplicity of oncology care, regular OTT board meetings can increase the accuracy of MDT’s work and treatment decisions without any overwork of the related disciplines and can also serve as an additive/alternative teamwork forum in the adjuvant, multiple line, and palliative care settings.

## Graphical Abstract

Graphical summary of the manuscript.

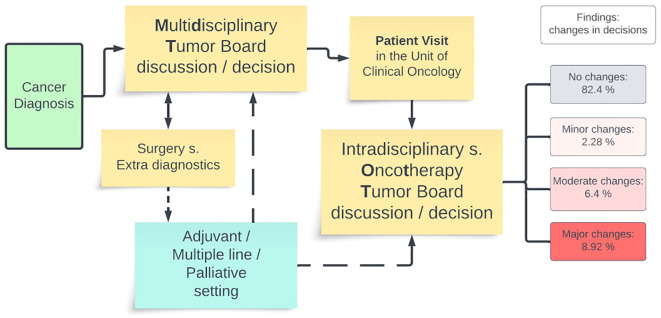

## 1 Background

Due to the ground-breaking development in both diagnostic and treatment methods, and the broad implementation of evidence-based and personalized medicine, cancer care has significantly improved over the last decades. Making the best treatment decisions with matched treatment delivery can lead to the most optimal treatment outcomes in both primary cancer care and in the management of relapsed and/or advanced disease states.

Multidisciplinary team (MDT) or tumor board discussions are generally accepted and guideline recommended forums for choosing the optimal treatment for cancer patients in the respective states of their disease. In the past decades, discussion-based decision making in MDTs has become a worldwide accepted quality control method and standard of patient care in almost every oncology unit (1–4). Nevertheless, to date there is an ongoing discussion about the substantive role of MDTs in cancer care. Their impact on survival data, the adherence level to their primary decisions, the role of case selection procedures, the cost- and time-effectiveness, have all been discussed (5–11). Furthermore, the increasing number of cancer patients, broadening spectrum of treatment options and multiple line treatments can both lead to an enormous rise in the workload of MDTs in different oncology centers. These challenges propose the necessity for periodically reconsidering the everyday work of the MDTs mainly in centers where the continuous multidisciplinary supervision is difficult to implement.

MDTs usually consist of different expert members, such as oncology surgeons, internal medicine experts (like pulmonologists, gastroenterologists, hematologists) and radiologists, pathologists, medical oncologists, radiotherapists, palliative care specialists, psychologists, cancer care nurses, social workers etc. Maintaining this form of top-level teamwork requires professional organization and it leads to great expense. However, the final treatment decisions generally reflect the specialty and the opinion of the dedicated expert members. Medical/clinical oncologists are generally considered to be one of the core members of MDTs, since they have the specialized knowledge related to the interpretation of clinical studies, dedicated treatment methods, molecular oncology and they participate in the life-long care of the patients (12).

As a result of the increasing number of patients and the complexity and multiplicity of their treatments over the previous years, we introduced a substantial change in the oncology decision making process in our tertiary cancer center. We hypothesized that the initial MDT-based oncotherapy treatment decisions could justify and individualize in a second step intradisciplinary or oncotherapy team (OTT) discussion, having the inevitable knowledge about patient’s preferences and tolerance, after an outpatient oncology consultation. Additionally, it seemed to be more time and cost efficient to discuss the different oncotherapy options exclusively by oncology experts in the purely adjuvant, multiple-line metastatic and palliative care settings.

Therefore, we established a two-step tumor board system at our tertiary cancer center in 2007. Over the conventional and organ-specific MDT discussions, we introduced additional OTT meetings, with the exclusive participation of medical oncologists, radiotherapists, and palliative care experts (13, 14). We hypothesized that this kind of teamwork would be appropriate for discussing the details of MDT’s oncotherapy recommendations with the aim of determining the items of different treatment regimens after the personal meetings with the patients and relatives. Moreover, we speculated that there was no absolute need for the continuous participation of different non-oncology experts when deciding on specific oncology issues.

Conclusively, the objective of our study was to introduce the work of the two-step tumor board system, to present our cancer treatment epidemiology experiences and particularly to analyze the decision outcomes of the OTT discussions, through retrospective real-world data analysis.

## 2 Materials and methods

### 2.1 Brief description of the two-step tumor board system

**Figure 1:**
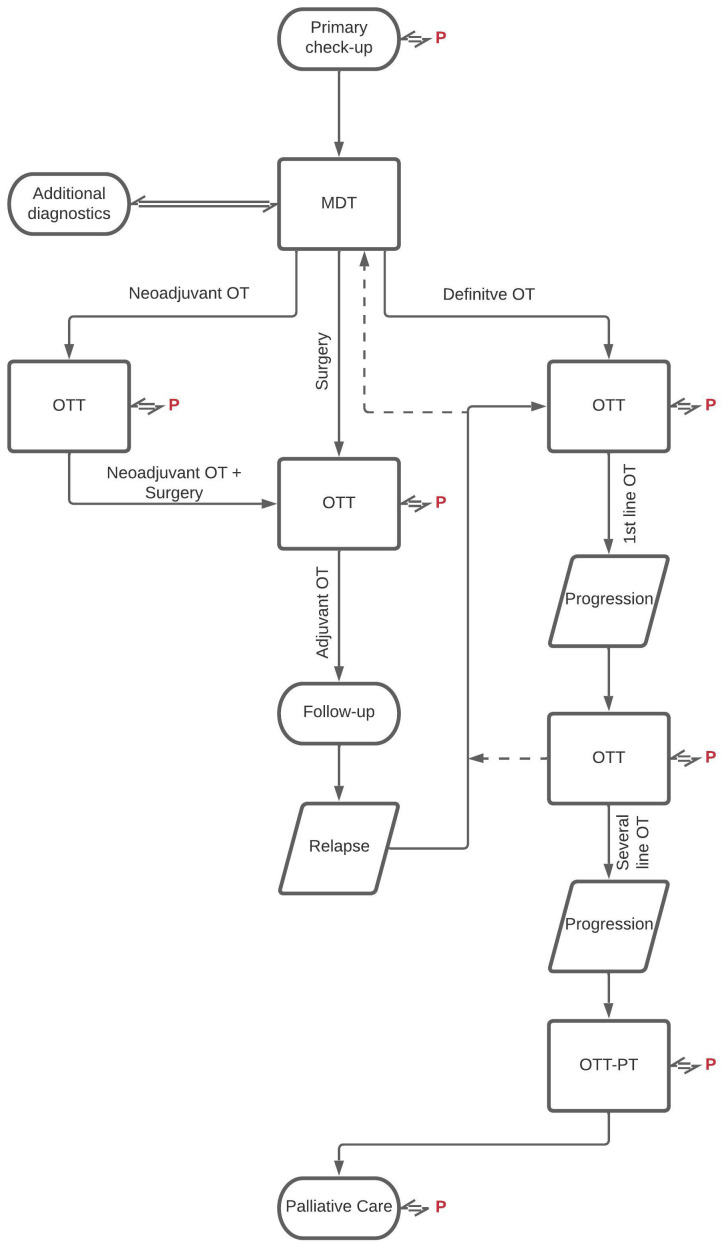
Workflow of the two-level tumor board system. OT, oncotherapy; MDT, multidisciplinary tumor board; OTT, oncotherapy team; PT, palliative team; P, patient.

Between 2007 and 2019 our tertiary oncology center was responsible for the cancer care of all solid malignancies (excluding the medical treatments of skin, lung, and gynecology cancers, but including the complex oncology care of brain, head and neck, breast, gastrointestinal, soft tissue, and urology cancers) for about a half a million population across the Southern Transdanubian region, in Hungary. The details and rules of the whole tumor board system were thoroughly planned and were closely monitored following its establishment in 2007 (13). The tasks of the different organ-specific MDTs continued to be carried out as usual, while the tasks of the OTT were explicitly determined. The recommendations of the MDTs were particularly discussed and confirmed again in the OTT meetings, while in purely adjuvant or multiple line settings repeated MDT was not automatically initiated, so in absolutely non-surgical cases we accepted the decision of the OTT discussion (**Figure 1**). Personal meeting with the patients always preceded the OTT discussions.

The OTT consisted of generally 8–12 oncotherapy specialists, medical oncologists, radiotherapists, and palliative care experts with the participation of pharmacists, study coordinators, psychologists, as oncology advocates. It should be emphasized that, to decrease the overwork of the MDT’s non-oncology members, in the second-level or OTT meetings only oncology experts (an intradisciplinary board) discussed the details of the different oncotherapy options for the patients, without the obligatory participation of interdisciplinary MDT team members comprising of pathology, radiology, surgery etc. experts.

OTT meetings were held 3 times a week, so there was no real waiting time for OTT meetings in the clinical practice (while organ-specific MDTs were held generally with 1-2-week intervals). The administration of the OTT was analogue to MDT meetings. Relevant clinical data, including patient history, comorbidities, general/performance state, histology, results of staging/restaging examinations, previous treatments etc. are separately documented in a specialized electronic medical registration system. The relevant medical documentation and a potential treatment plan based on MDT recommendations were registered before the OTT meeting by the treating physician, containing some basic information about the probable tolerability of the treatment and the patient preferences. During the OTT meeting, the physicians discussed the treatment plan, accepted the original plan, amended, or modified it. Like the MDT meeting workflow, relevant data and decisions were recorded in the medical registration system and all the participants were required to sign the final document.

### 2.2 Data collection and elaboration

Based on the OTT data previously recorded in the medical registration system of the University of Pécs (eMedSolution HMS) we established an anonymized electronic dataset for further analysis. We collected all available items about the diagnosis, the age of the patient, the type of final treatment, the number of previous discussions s. oncotherapy treatment lines on the same patient, finally the differences between treatment recommendations and decisions. We recorded all the data/modifications above.

We analyzed retrospectively the OTT documentation from a 12-year period, between November 2007 and November 2019. We completed data collection before the COVID pandemic period since the tasks of our tertiary cancer center had notably changed in those times. Relevant patient history data, treatment plan, and treatment decision data were separately aggregated and after accurate determination of professional phrases, a detailed analysis was performed using a regular expression-based word search algorithm. If there was any doubt in the results we overviewed individually the patient’s data. Treatment types (radiotherapy, radio-chemotherapy, chemotherapy, endocrine replacement therapy, targeted therapy, immunotherapy, combination treatments or additional diagnostic/surgical intervention, observation, best supportive care) were identified based on the OTT’s final decision.

To control the accuracy of the treatment plans, four variance categories were established. These categories were as follows: 1., acceptance of the original option “without modification”, 2., “minor modification”, when the treatment plan was considered eligible, but some additional suggestions were made (e.g., extra consultation, intensified supportive care or follow-up, etc.) or there was a negligible (less than 10%) difference in the final radiotherapy (RT) dose or in the number of recommended chemotherapy cycles, 3., “moderate modification” when the treatment method was distinctly changed (e.g., RT dose modification over 10%, omission or addition of a chemotherapy component, etc.), and 4., “major modification” when the choice of treatment was completely transformed (e.g. surgical consultation/intervention over any kind of oncotherapy, stereotactic body radiotherapy instead of any systemic therapy, targeted therapy based treatment over conventional chemotherapy, or radio-chemotherapy instead of RT or vice versa). Those cases were particularly included in the data elaboration, where treatment plans were not properly recorded in the registration system, which mainly occurred when less-experienced colleagues were the treating physicians.

### 2.3 Data analysis

One-year long periods (based on calendar years) were defined and analyzed, assessing the number of OTT decisions, the age distribution of the patients, the volume of several treatment decisions/lines in one patient, the ratio of modern targeted and immunotherapies and the data from the treatment modification categories. For some statistical analysis, four-year periods (2008–2011 *vs*. 2012–2015 *vs*. 2016-2019) were generated, and the comparison was made between the first and the last four-year periods using paired sample T-tests. Considering the number of intra-patient OTT decisions, five-year periods were defined from the year of theoretical equilibrium, 2010, supposing a median 3 years for the palliative treatment period of metastatic diseases. Statistical analyses were performed only in some nominated epidemiological parameters (number of OTT cases, patient’s age, number of treatment lines etc.).

## 3 Results

**Figure 2:**
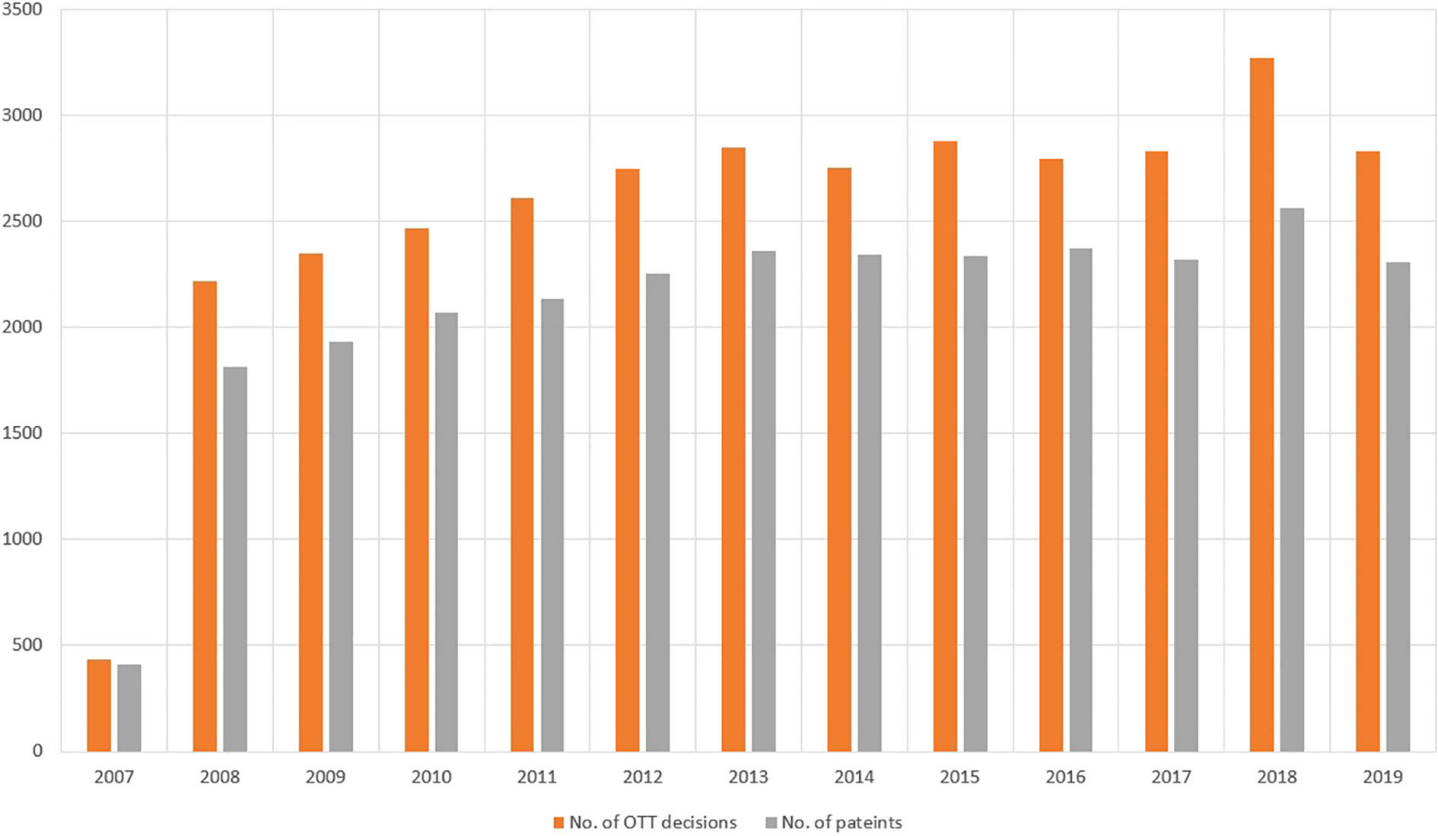
The number of OTT discussions (red bars) and number of patients (gray bars) between Nov. 2007 and Nov. 2019 at the Institute of Oncotherapy, University of Pécs. Considering both patient and case numbers, a continuous increase can be observed, with p values of 0.0021 and 0.0035, comparing the data of the first and the last 4-year periods.

Altogether, 33,056 cases of 27,227 patients were discussed in the second level OTT decision system during the 12-year study period. There was an annual increase of 1815 to 2566 in the number of patients, and of 2221 to 3269 in the number of OTT discussions between 2008 and 2018, indicating a 41% and 47% rise, respectively (**Figure 2**). Comparing the periods of the first and the last 4 years, the increase was significant considering both the number of patients (p=0.0021) and the number of discussed cases (p=0.0035).

Concerning the age distribution of the patients, remarkable changes could be observed during the 12-year period. The number of patients over 60 years of age increased significantly, the percentage increase was 26.3 to 37.0%, 20.2 to 26.6%, and 1.2 to 8.1%, in the 60-70, 70-80, and over 80 years groups, respectively. Comparing the first and the last 4 years the increment in both analyzed age categories (between 60 and 80 years, and over 80 years), was significant, with p values of 0.0019 and 0.0011, respectively.

The distribution of the OTT meetings decisions between treatment regimens were as follows: radiotherapy 32.7%, chemotherapy 24.6%, radio-chemotherapy 7.6%, endocrine replacement monotherapy 3.1%, targeted therapy 3.2%, pure immunotherapy 0.2% (please note that in those times the systemic treatment of melanoma and lung cancer belonged to another departments), and any forms of treatments using combined modalities 16.9%. Novel diagnostic/surgical interventions, “watch and wait” observation or best supportive care were recommended in 11.7% of the cases discussed. Considering the most sophisticated treatment options, a rising tendency could clearly be observed concerning targeted treatments and immunotherapies (based on the evolution of molecular analyses). Between 2008 and 2018 the percentage of these modern therapies increased from 7.1% to 12.9%, considering both monotherapies (3.2% to 4.4%) and combinations with other therapies (3.9% to 8.5%).

**Figure 3:**
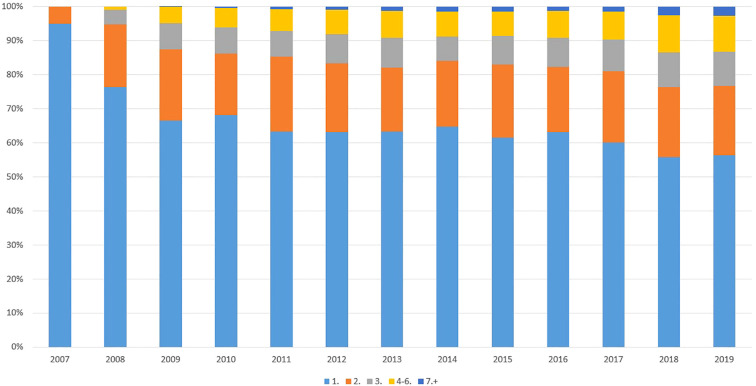
Ratio of OTT decisions regarding the same patient in the study period. The average number of oncotherapy tumor board (OTT) discussions for the same patient continuously increased over the years.

There was an inevitable increase in the number of intra-patient OTT decisions/treatment lines, as well (**Figure 3**). Considering the data from year 2010 (year of equilibrium) and year 2018, the percentage of 2^nd^, 3^rd^, 4-6^th^ and 7^th^+ decisions were 18.0, 7.5, 5.8 and 0.4% *vs*. 20.7, 10.2, 10.9 and 2.5%, respectively. Comparing the ratios of the 2010–2014 and 2015–2019 periods and the 3^rd^, 4-6^th^ and 7^th^+ decisions, the changes were significant, with p values of 0.024, 0.0141 and 0.002, respectively.

Conclusively, the increased number of patients and the discussed cases, the mean age increments, and the increased number of intra-patient OTT decisions all show evidence for the increasing complexity, multiplicity and effectiveness of cancer care during the 12-year observation period.

**Figure 4:**
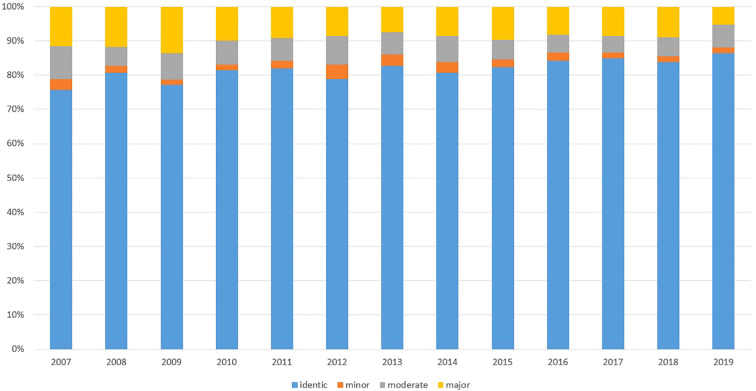
Ratio of decision changes in OTT discussions. The 12-year averages of minor, moderate and major alterations in decisions were 2.28, 6.4 and 8.92%, respectively. The continuous decrease in the ratios of decision modifications can be explained as a “team learning effect”.

Finally, to validate our basic hypothesis, that the second-level OTT meeting can unequivocally modify the original treatment recommendations, we analyzed the extent of treatment decision modification in OTTs (**Figure 4**). During the 12-years observation period the percentage of the non-applicable data (without treatment plan recommendation) unambiguously decreased (26.04 to 7.09%). The percentage of modified decisions decreased as well (24.3 to 13.56%), also supporting a team learning effect. Nevertheless, the average ratio of the minor, moderate and major changes were 2.28, 6.4, 8.92%, respectively. Conclusively, a second overview fundamentally altered (with moderate and major modifications) cancer therapy in cc. 15%.

Considering only the first decisions of the OTT, the average ratio of the minor, moderate and major changes were 2.63, 5.84, and 7.83%, respectively. Conclusively, albeit to a lesser extent, the first MDT-based treatment decisions were also modified by the OTT in primary care, as well, potentially due to the previous meeting with the patients. Considering only the second and the third decisions the changes were 1.83, 7.14, 10.61% and 1.26, 7.5, 11.28%, respectively.

We separately analyzed the decision modifications in different types of cancer as well, and considering only the frequent cancer diseases, interestingly the most pronounced minor, moderate and major decision modifications were observed in cervical (5.65, 10.82 and 10.59%), ovarian (2.00, 9.33 and 12.67%), bladder (6.47, 5.76 and 10,07%), breast (1.28, 6.45 and 14,44%), and biliary tract (2.59, 5.17 and 14.44%) tumors, respectively.

Analyzing the 2^nd^, 3^rd^, and 4^th^ plus decisions of the OTT, the average ratio of minor, moderate and major modifications were 1.65, 7.4, 10.88%, 1.44, 7.72, 10.2%, and 1.62, 7.93, 11.12%, respectively. Learning from all these data, a relatively greater number of moderate and major treatment modifications could be observed in the multiple line treatment settings (**Table 1**), presumably due to only OTT-based workflow in the palliative treatment period and the absence of high-level evidence in multiple line settings.

**Table 1:** Minor, moderate, and major treatment decision modifications in all OTT cases without missing data (No. 30,183), considering the number of the decisions for the same patients.

All OTT decisions			
No. of treatment line/decision	Minor changes (%)	Moderate changes (%)	Major changes (%)
First	2.63	5.84	7.83
2^nd^ plus	1.65	7.4	10.88
Second	1.83	7.14	10.61
3^rd^ plus	1.44	7.72	10.2
Third	1.26	7.5	11.28
4^th^ plus	1.62	7.93	11.12
Total	2.28	6.4	8.92

## 4 Discussion

The history of MDTs started with the first description of a tumor board meeting in 1952 when Bell and colleagues established this type of medical conference in a children’s hospital, in Washington DC. To our knowledge the first tumor board proceeding was published in 1968 by Bottomley, and the first documentation of multidisciplinary teamwork was published by Murphy (15–18). Since then, MDTs have gradually become a routine part of clinical work in oncological care, worldwide. The original aim of MDT meetings was to determine the direction of the first definitive treatment, like surgery, radiotherapy, or chemotherapy, but later MDTs also attempted to determine the other elements of complex cancer care.

Although the undeniable role of MDTs in cancer care is generally accepted, and a great number of studies described changes in staging, diagnosis, initial management, and higher adherence to clinical guidelines, only a few authors have succeeded in demonstrating the positive effects of MDTs on patient survival rates (1, 4, 8, 19, 20). Nevertheless, the significance of MDTs in dividing responsibility among team members, in enabling teamwork, in providing a legal framework for medical decision-making and for enhancing education opportunities have been generally emphasized (1–9, 21, 22).

Numerous studies have described the work of different organ or disease-specific MDTs, like head and neck cancer, breast cancer, brain tumor, lung cancer, gastrointestinal tumor etc. boards (3, 19, 23–28). Reviews assessing the general judgment profiles, and the overall advantages and disadvantages of MDTs have also been published. Prades et al. (1) analyzed 51 peer-reviewed papers published between 2005 and 2012 to determine the pros and cons of MDTs. The article concluded that MDTs were associated with relevant changes in clinical diagnostic and treatment decisions in most tumor types and that MDTs led to better clinical outcomes in several tumor types, like colorectal, head and neck, breast, and lung cancer.

By assessing five systematic reviews, Specchia et al. (20) concluded that the multidisciplinary approach is the best way to deliver complex cancer care. The effects of the decisions of MDTs were analyzed regarding diagnosis, treatment form, waiting times, quality of life, satisfaction, recurrence score, survival, extra visits to general practitioners among others. MDTs led to unambiguous changes in diagnosis, treatment, and in some survival respects. Rosell et al. (29) aimed to estimate the benefits and limitations of MDT meetings for health professionals in the Swedish cancer care system. The authors ranked the support for patient management, competence development, to a lesser extent the monitoring of patients for clinical trial inclusion, and accurate treatment recommendations, and high-level adherence to clinical guidelines as benefits of MDT meetings. In a recent summary of the MDT approach, Berardi et al. (6) stated the higher adherence to protocols and guidelines, possibly better clinical outcomes, and development in decision making processes as its advantages, but concluded that the costs of the work, some legal responsibility issues, geographic and travel difficulties, and in some cases the treatment delays could be negative effects of MDT work. Hahlweg et al. (30) assessed the quality of decision-making by analyzing the quality of case history, radiological and psychosocial information, data about comorbidities, patient views and the number and behavior of participants and quality of work. According to this investigation, MDTs have high-quality cancer-specific medical information and low-quality information on patient views and psychosocial information. They also found that time constraints have an inevitable role if the MDT had made patient-centered decisions. Wihl et al. (31, 32) also accentuated the under-representation of eligible patient related and non-medical data in MDTs.

The basic aim of our work was to assess the treatment plan modification ratios during OTT discussions, so the comparison of our results to the experiences of other authors is inevitably important. In the basic review of Pillay et al. (8), 4–45 percent changes were found in the final decisions of MDTs, nevertheless, patients whose histories were discussed in MDT meetings generally received a more accurate treatment regime. Other studies also found similar modification rates considering original treatment plans and MDT’s treatment decisions. For example, in their review, Lamb et al. (33) reported 2-52% treatment modifications in different studies, while Freytag et al. (34) demonstrated the positive effect of multiple MDTs on patient survival that was partially explained by the repeated additional overview of the cancer history of the patients. However, to our current knowledge, there is no sufficient data available about the ratio of treatment modifications in multiple-line treatment settings. Returning to the work of Freytag, the repeated/multiple tumor board discussions had inevitably a positive effect on the outcome and this finding seems to be justified in our efforts. Considering our system, the patient-related medical and psychosocial information was strictly integrated in the OTT decisions, ensuring the evolutive role of repeated overview of patient data and treatment decisions. We experienced cc. 12-18% substantial (major + moderate) modification of the original treatment plan during OTT, considering primary care, first-line and multiple-line treatment settings.

Adherence to the professional team decision following an MDT meeting remains a constant question. Previously, several studies have reported a great difference between the recommendations made by the MDTs and the subsequent real-life care of the patients. Lamb et al. found that decisions could not be implemented in 1-16% of the cases (33). According to Blazeby et al. (35) 15.1% of the decisions were discordant and not implemented, mainly due to co-morbid health issues, patient choice and the absence of eligible clinical information. According to Hollunder et al. (36), only 80.1% of all MDT’s decisions were fully implemented and 8.3% of all recommendations were substantially modified in actual clinical practice. The most common reasons for these changes were patient wishes, early patient deaths and decisions of the individual physicians, based on the patients’ comorbidities or anticipated serious side effects of the recommended treatment. It was concluded that there was a need for document optimization, increased consideration of patients’ preferences and patient-related data and sufficient patient contact before the MDT. However, the actual participation of the patient in MDTs could sometimes impede and sometimes improve the decision-making process (11, 37, 38). From the patients’ point of view, in the analysis of Anshan et al. (39), most of the patients reported positive experiences and recommended participation in MDTs, but some patients regretted the active participation. However, the patient always has the right to refuse the decision (40).

Nevertheless, considering the significance of patient-related data, treatment modification rates and adherence to the MDT’s decision, it should be emphasized that the first personal meeting between the patient and the treating oncologist (or surgeon) is generally organized after the MDT discussion in most cases. Our system differs from the general practice, since an outpatient oncology consultation is an obligatory criterion of admission to OTT. Although we did not monitor the adherence to our second-level OTT recommendations, we assume that the proportion of treatment refusals/failures was negligible. Our medical oncology and radiotherapy experts participating in the OTT were acquainted with the patients’ characters, compliance, general state, comorbidities, tolerance, and own views through personal meetings with the patients and the relatives prior to the OTT decision. Therefore, the feasibility of the treatments was considered previously, based on both the patients’ preferences and on the knowledge and experience of specialists in oncology. Unfortunately, we have no data about patient satisfaction and the possible changes in their quality of life.

Due to the spectacular development and expanding of cancer therapeutic options, their expenses, the new types of side effects and the consecutive challenges of selecting the most optimal therapy, new standards of MDTs such as molecular and immunological tumor boards have been established (41–47). The establishment of the molecular boards is another answer to the increasing complexity of cancer care; however, it is generally applicable mainly in academic cancer centers.

Alongside the inevitable development of systemic therapies, the spectrum of local treatments has also changed. Options for the successful combination of local and systemic treatments have also increased, and with the development of radiotherapy facilities, stereotactic body radiotherapy (SBRT) has become a part of MDT discussions and everyday practice (48–50). Finally, some studies emphasize the significance of MDT meetings in the proper screening of patients for oncology trials (6, 25, 29, 51). In summary, the routine application of all the modern treatment technologies above, including targeted agents, immunotherapy, SBRT, combined oncological modalities and clinical trials, necessitate special oncological knowledge and experience. It should be highlighted that these modalities can be utilized in several lines throughout the patient’s cancer disease history. Nevertheless, our descriptive epidemiology data with the increasing number of discussed cases mainly due to the multiple line treatments and their efficacy and with the increasing average age of the patients and the necessity of cautious treatment choices both prove the increasing complexity of cancer care.

To overcome this complexity of cancer care, recently other reports have emphasized the necessity of improved documentation, implementation of digital technology throughout the MDT process, and the potential introduction of artificial intelligence or different clinical decision support systems into the MDT decision-making process (22, 23, 43, 52–56).

Returning to the basic work of Popescu et al. (12), the medical/clinical oncologist is considered one of the core members of the MDT providing a comprehensive approach to cancer care. Medical oncologists have knowledge regarding both scientific and clinical evidence, clinical trials, safety, comorbidities, quality of life and cost-effectiveness “through the entire cancer journey”. To support this theory and the special role of medical oncologists in MDTs Popescu emphasized the rapidly growing number of cancer patients and the innovations, and he also suggested the need for continuous education. In accordance with these observations, other authors also have also emphasized the special role of medical oncologists in MDTs (35, 57, 58), moreover the fashion of the MDT’s leadership in the treatment decision (59). However, Valentini et al. (60) accentuated the significance of real multidisciplinary cooperation as opposed to the meetings dominated by medical oncologists.

Based on our original hypothesis and our OTT experience we believe that clinical oncologists have fundamental tasks particularly in the adjuvant and metastatic settings of cancer therapies. Furthermore, our OTT strategy offers a good opportunity for educating oncology residents, shares responsibility in decisions and assists patients with advanced cancer to enter palliative care (61, 62).

Strengths and limitations of our work and experiences: to our knowledge this is the first big data elaboration concerning an intradisciplinary tumor board system operation, and teamwork effect on multiple-line oncotherapy’s. The main limitations of the project are the missing data about the adherence and the outcome effect of the intradisciplinary team decisions, as we have no data elaborated about patient satisfaction and the time-effect of extra meetings. However, within the framework of the grant supporting this project, we assessed the real-life survival probabilities of metastatic colorectal cancer patients in the same period, and we found that our treatment results correspond to the literature data (63, 64). Another limitation of the work is that it is only a single-center experience, and exclusively a retrospective historical data comparison was carried out.

Considering the outcomes of our study, it is already well known that MDT meetings play a crucial role in decision making for the treatment of all cancer patients, allowing better communication, coordination and decision-making among healthcare professionals when evaluating different treatment options. In addition, here we succeeded in presenting that a second step OTT discussion with the participation of a greater number of clinical oncology experts could modify the original treatment plans. We succeeded in proving that the complexity of cancer care had continuously improved, with the increasing number of the treatment decisions and tasks considering every single cancer patient. Another new finding of the present study is that the OTT meetings can complement the original MDT board decisions and potentially deputize them in the purely adjuvant and in the multiple line settings, reducing the workload of multidisciplinary teams by at least 30-40% (see **Figure 3**). Nevertheless, it should be emphasized that our system cannot replace the well-functioning multidisciplinary tumor board discussions, but it can provide an alternative solution in medium- and low-income regions/countries, especially in centers that treat different tumor entities and have a limited number of specialized experts.

## 5 Conclusions

Teamwork, the sharing of knowledge and responsibility is a fundamental part of cancer care. Nevertheless, considering limited professional human resources, the inevitable progress of medicine and technology, as well as legal-, social- and financial issues, there is a need for the improvement in teamwork of cancer care, adaptable to different level oncology centers. Our special approach to this quintessential question was the establishment of the intradisciplinary oncotherapy tumor board (OTT) system. We also believe that this type of quality control could be a step further to the individualized cancer care with maximal respect to the treatment requests and the tolerance of the patients. Nevertheless, OTT is not an absolute substitute for MDT, but it can be a potential and partial alternative in certain healthcare systems or in certain healthcare situations. Concluding, the complexity of cancer care and the increasing number of cancer patients requires further development of the decision-making systems with the utilization of advanced teamwork and artificial intelligence, and with special knowledge of evidence-based medicine and real-world experiences.

## Data Availability

The raw data supporting the conclusions of this article will be made available by the authors, without undue reservation.
